# 16S rRNA Gene Amplicon Data Set-Based Bacterial Diversity in a Water-Soil Sample from Pangong Tso Lake, a High-Altitude Grassland Lake of the Northwest Himalayas

**DOI:** 10.1128/MRA.01192-18

**Published:** 2018-11-01

**Authors:** Garima Bisht, Anuradha Sourirajan, David J. Baumler, Kamal Dev

**Affiliations:** a Faculty of Applied Sciences and Biotechnology, Shoolini University, Solan, Himachal Pradesh, India; b Department of Food Science and Nutrition, University of Minnesota—Twin Cities, St. Paul, Minnesota, USA; c Microbial and Plant Genomic Institute, University of Minnesota—Twin Cities, St. Paul, Minnesota, USA; d Biotechnology Institute, University of Minnesota—Twin Cities, St. Paul, Minnesota, USA; Georgia Institute of Technology

## Abstract

We report here 16S rRNA-based bacterial diversity existing during freezing conditions in a high-altitude Himalayan lake through sequencing a 16S rRNA gene amplicon data set. A total of 121,857 high-quality reads were obtained; 40.78% of the bacterial population was classified to the genus level, while 1.26% was classified to the species level.

## ANNOUNCEMENT

**FIG 1:**
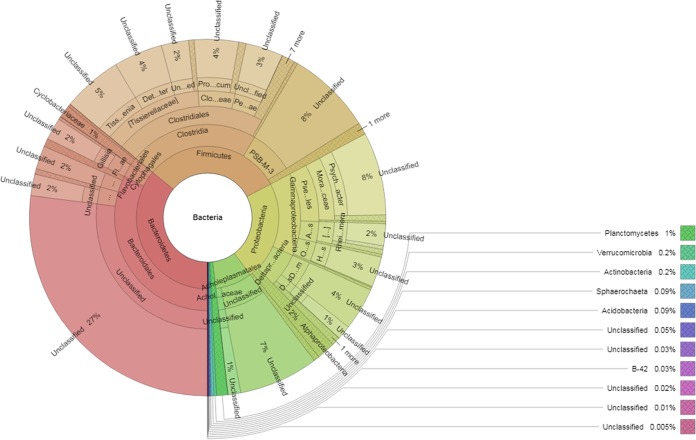
Krona chart of the bacteria represented by 16S rRNA gene amplicon-based bacterial diversity in a soil-water sample from a high-altitude cold desert lake. Each circle represents the phylum, class, order, family, genus, and species from the inside to the outside of the circle, respectively, indicated by percent diversity based on the absolute number of representative bacteria.

High-altitude lakes in the Himalayas represent unique areas of microbially biodiverse populations that are exposed to freezing temperatures, salinity, high UV radiation, low oxygen, and limited nutrients (1). Pangong Tso Lake (33.819770ʺN, 78.614501ʺE) is one such lake, situated at an elevation of about 4,350 m in the Northwest Himalayas. Metagenomic studies of such unique environments are gaining importance in providing a better understanding of microbial ecology and its applications to biotechnology (2–4). Unlike previous studies which explored the whole metagenome in a sample collected in the month of September (5), we collected a water-soil mixture (pH 9.0) aseptically underneath the frozen lake surface (1 foot depth; ice was broken to create a hole) using a sterile long-handled stainless steel ladle during the peak of winter (January; temperature of –10°C) to specifically identify bacterial diversity. The salinity of the water-soil slurry was determined by quantitation of ions using flame photometry (6). The water-soil slurry contained 29, 0.6, and 14.73 mmol per liter of sodium, potassium, and chloride ions, respectively. The total salinity of the water-soil slurry was ∼0.12%. The total genomic DNA was isolated from the water-soil mixture using a Nucleospin soil kit (TaKaRa Bio, Ltd.), and an *A*_260/280_ of ∼1.81 was quantified with a NanoDrop instrument. The amplicon library was prepared using a Nextera XT index kit (Illumina, Inc.). The primers for the amplification of the V3-V4 region of 16S rRNA genes (forward, GCCTACGGGNGGCWGCAG, and reverse, ACTACHVGGGTATCTAATCC) were designed at Eurofins. The V3-V4 region is highly variable and known to reveal microbially diverse populations (7). The amplicon library was purified using 1× AMPure XP beads, quantified using a Qubit fluorometer, and analyzed with a 4200 TapeStation system (Agilent Technologies) using D1000 screen tape following the manufacturer’s instructions. The library was loaded onto a MiSeq instrument (2 × 300 bp) at 10 to 20 pM for cluster generation and sequencing. The sequenced raw reads were processed to obtain high-quality (HQ) reads using Trimmomatic v0.35 (8) to remove adaptor sequences, ambiguous reads, and low-quality sequences (i.e., those with a more than 10% quality threshold [QV] of <20 Phred quality score). A total of 121,857 high-quality reads were obtained. The HQ reads were subjected to operational taxonomic unit (OUT) identification at 97% sequence similarity and taxonomic assignment of OTUs using the Greengenes database (16S/Archaea database) and the Quantitative Insights into Microbial Ecology (QIIME) module. Krona-based (9) diagram visualization showed that 99.22% of the microbially diverse population represents *Bacteroidetes* (35.97%), *Firmicutes* (31.27%), *Proteobacteria* (22.56%), *Tenericutes* (8.38%), and *Planctomycetes* (1.04%) at the phylum level (Fig. 1). The remaining microbially diverse population represents the phyla *Verrucomicrobia* (0.23%), *Actinobacteria* (0.23%), *Spirochaetae* (0.09%), *Acidobacteria* (0.09%), *Cyanobacteria* (0.05%), *Gemmatimonadetes* (0.03%), and *Deinococcus-Thermus* (0.03%) and the candidate phyla TM6 (0.02%) and SR1 (0.01%). A fraction of the population (0.05%) was unclassified even at the phylum level. Only 40.78% of the bacterial population was classified to the genus level, and 1.26%, representing *Firmicutes* (0.209088%), *Proteobacteria* (0.471663%), and *Bacteroidetes* (0.585932%), was classified to the species level (Table 1). About 2.70025771% and 0.068075174% were represented by members of the genera *Halomonas* and *Marinomonas*, respectively.

**TABLE 1:** Summary of bacteria identified to the species level from a 16S rRNA gene-based metagenomic study of a soil-water mixture from a high-altitude lake

Phylum	Class	Order	Family	Species	Absolute count	%
*Firmicutes*	*Clostridia*	*Clostridiales*	*Clostridiaceae*	*Alkaliphilus transvaalensis*^*a*^	3	0.0072
	*Clostridia*	*Clostridiales*	*Peptostreptococcaceae*	*Clostridium sticklandii*^*b*^	4	0.0097
	*Clostridia*	*Clostridiales*	*Peptococcaceae*	*Desulfosporosinus meridiei*^*c*^	28	0.0680
	*Bacilli*	*Bacillales*	*Planococcaceae*	*Planococcus pelagicus*^*d*^	51	0.1240
*Proteobacteria*	*Betaproteobacteria*	*Methylophilales*	*Methylophilaceae*	*Methylotenera mobilis*^*e*^	25	0.0608
	*Gammaproteobacteria*	*Alteromonadales*	*OM60*	*Haliea mediterranea*^*f*^	4	0.0097
	*Gammaproteobacteria*	*Oceanospirillales*	*Oceanospirillaceae*	*Marinomonas primoryensis*^*g*^	5	0.0122
	*Gammaproteobacteria*	*Alteromonadales*	*Chromatiaceae*	*Rheinheimera perlucida*^*h*^	138	0.3355
	*Alphaproteobacteria*	*Rhodobacterales*	*Rhodobacteraceae*	*Paracoccus marcusii*^*i*^	22	0.0535
*Bacteroidetes*	*Cytophagia*	*Cytophagales*	*Cyclobacteriaceae*	*Aquiflexum balticum*^*j*^	6	0.0146
	*Cytophagia*	*Cytophagales*	*Cyclobacteriaceae*	*Rhodonellum psychrophilum*^*k*^	235	0.5713

a Extremely alkaliphilic.

b Anaerobic motile Gram-positive bacterium, nonpathogenic to humans.

c Sulfate-reducing bacterium.

d Rare isolate.

e Methylamine-utilizing bacterium.

f Halophile, pale yellow pigmented.

g Gram-negative, aerobic, psychrophilic, halophilic, and motile bacterium.

h Marine bacterium.

i Orange Gram-negative coccus.

j Marine bacterium isolated from the Baltic Sea.

k A psychrophilic and alkaliphilic bacterium reported in the literature and a pure isolate from soil-water mixture in the current study is reported as *Rhodonelum psychrophilum* strain GL8 under GenBank accession number MH031708.

### Data availability.

The 16S rRNA gene amplicon data set is available at NCBI (National Center for Biotechnology Information) under the SRA accession number SRP158149.
